# Adult-recognized 22q11.2 deletion syndrome in adult neurology practice: a brief report of two diagnostic pitfalls

**DOI:** 10.3389/fneur.2026.1933196

**Published:** 2026-08-31

**Authors:** Daren Wu, Yuemiao Wang, Dandan Sun, Jiawei Wang, Xun Wang

**Affiliations:** 1 Anhui University of Traditional Chinese Medicine, Hefei, China; 2 Department of Neurology, The Affiliated Hospital of the Institute of Neurology, Anhui University of Chinese Medicine, Hefei, China

**Keywords:** 22q11.2 deletion syndrome, adult neurology, basal ganglia calcification, diagnostic pitfall, parkinsonism-mimicking syndrome, pyramidal syndrome

## Abstract

**Background:**

22q11.2 deletion syndrome (22q11.2DS) may be overlooked in adults when neurological care is prompted by pyramidal, gait, or parkinsonism-mimicking presentations rather than by congenital, developmental, psychiatric, or systemic features.

**Methods:**

We retrospectively reviewed the clinical, imaging, and genetic findings of two unrelated adults whose neurological presentations led to whole-exome sequencing-based copy-number variant (CNV) detection of 22q11.21 deletions. Orthogonal confirmation by chromosomal microarray, multiplex ligation-dependent probe amplification (MLPA), quantitative polymerase chain reaction (qPCR), fluorescence *in situ* hybridization (FISH), or copy-number variation sequencing (CNV-seq) was not available; therefore, the reported deletion sizes and coordinates represent WES-derived estimates.

**Results:**

Patient 1 presented with progressive lower-limb weakness, a spastic unsteady gait, pyramidal signs, dysarthria, and ataxic features, together with learning difficulty, palatal abnormalities, and a maternal history of similar gait disturbance and suspected epilepsy; analysis identified a 2.67-Mb deletion at 22q11.21. Patient 2 presented with progressive limb weakness, dysarthria, parkinsonism-mimicking rigidity and slowness, bilateral basal ganglia calcification, abnormal globus pallidus MRI signals, developmental delay, psychiatric symptoms, craniofacial features, and a maternal history of similar motor and cognitive impairment; analysis identified a 2.52-Mb deletion at 22q11.21.

**Conclusion:**

These cases highlight diagnostic pitfalls and syndromic clues rather than establish a new mechanism. In adult neurology practice, developmental history, palatal or craniofacial abnormalities, basal ganglia calcification, psychiatric symptoms, and family history should prompt consideration of 22q11.2DS and genetic testing that is sensitive to copy-number variants.

## 1 Introduction

22q11.2 deletion syndrome (22q11.2DS) is a multisystem copy-number variant (CNV) disorder with neurodevelopmental, psychiatric, craniofacial, palatal, endocrine, immune, and neurological manifestations. Adult patients may first present to neurologists with progressive gait disturbances, pyramidal signs, or parkinsonism-mimicking presentations, while childhood developmental or syndromic clues are subtle, remote, or not actively elicited. Published reviews and adult cohort data indicate that movement disorders, particularly parkinsonism, are increasingly recognized in 22q11.2DS (1–5). The clinical question in the present report is therefore not whether movement disorders can occur in 22q11.2DS, but rather which overlooked clues should redirect an apparently isolated adult neurological presentation toward syndromic CNV testing. Table 1 summarizes selected recent literature and places the present cases in the context of adult diagnostic-pitfall presentations. Unlike prior studies emphasizing adult parkinsonism in 22q11.2DS (1–4), the present report focuses on delayed syndromic recognition in adults presenting with hereditary spastic paraplegia (HSP)-like, spinocerebellar ataxia (SCA)-like, or Fahr-like neurological pathways, with developmental clues having been overlooked.

**Table 1:** Selected recent literature on motor phenotypes in 22q11.2 deletion syndrome and comparison with the present cases.

Study	Design/Patients	Main motor phenotype	Key diagnostic message	Diagnostic context for the present cases
Reyes et al. 2026 (1)	Scoping review; 43 eligible studies	Expanding 22q11.2DS movement-disorder spectrum, with parkinsonism emphasized	Movement disorders in 22q11.2DS are currently a recognized clinical spectrum rather than isolated curiosities	Shows that movement disorders in 22q11.2DS are already systematically recognized; the present report should not be framed as a new movement-disorder phenotype
Reyes et al. 2025 (2)	Adult deep-phenotyping study; 31 adults with 22q11.2DS and movement disorder	Non-parkinsonian tremor, parkinsonism, dystonia, myoclonus, dyskinesia, stereotypies, and functional movement disorder	Adult 22q11.2DS motor phenotypes are heterogeneous and frequently coexist with neuropsychiatric or developmental features	Demonstrates the complexity of adult motor phenotypes; the present cases add bedside recognition routes from pyramidal/HSP/SCA-like and calcification/Fahr-like presentations
von Scheibler et al. 2025 (3)	Multicenter prevalence study; 856 adults with 22q11.2DS	Clinically diagnosed Parkinson disease; reported a median motor onset at 45 years	22q11.2DS is associated with increased PD risk and relatively early motor onset	Establishes increased PD risk in adults with 22q11.2DS; Patient 2 is parkinsonism-mimicking but does not meet the criteria for confirmed PD
Boot et al. 2020 (4)	Adult case–control study; 82 adults with 22q11.2DS and 25 controls	Age-related parkinsonian signs including bradykinesia, rigidity, and rest tremor	Subclinical or early parkinsonian signs may be detectable in adults with 22q11.2DS	Shows that early or subclinical parkinsonian signs can occur in adults with 22q11.2DS; the present report focuses on diagnostic misdirection in symptomatic care
Piervincenzi et al. 2022 (8)	Adult imaging study; 29 adults with 22q11.2DS	Parkinsonism associated with cerebellar structural abnormalities	Cerebellar involvement may accompany 22q11.2DS-related parkinsonism	Supports cerebellar involvement in 22q11.2DS-related parkinsonism; Patient 1 had ataxic features but lacked quantitative cerebellar imaging evidence
Present cases	Two adults recognized in an adult neurology setting	Patient 1: pyramidal syndrome with ataxic features. Patient 2: parkinsonism-mimicking rigidity/slowness with basal ganglia calcification	Developmental history, palatal/craniofacial abnormalities, psychiatric symptoms, calcification, and family history redirected the diagnosis toward 22q11.2DS	Emphasizes two bedside traps: an HSP/SCA-like pyramidal presentation and a Fahr-like calcification pathway before 22q11.2DS is considered

22q11.2DS, 22q11.2 deletion syndrome; HSP, hereditary spastic paraplegia; PD, Parkinson disease; SCA, spinocerebellar ataxia.

## 2 Methods

### 2.1 Study design and genetic testing

This was a retrospective descriptive report of two unrelated adult patients evaluated in an adult neurology setting. Clinical history, neurological examination, imaging, routine laboratory findings, and available genetic reports were reviewed. The report was intended to describe diagnostic pathways rather than to test disease mechanisms or variant pathogenicity. Genetic testing was performed using whole-exome sequencing (WES) with copy-number variant (CNV) analysis. Peripheral blood DNA was captured using the IDT xGen Exome Research Panel v1.0 (39 Mb; Integrated DNA Technologies, Inc., Coralville, IA, USA; catalog number 1056115), and sequencing was performed using the Illumina NovaSeq 6000 platform with the NovaSeq 6000 S4 Reagent Kit v1.5 (300 cycles; Illumina, Inc., San Diego, CA, USA; catalog number 20028312). Read-depth data were corrected for GC and repeat-related biases, segmented using circular binary segmentation, and classified according to segment-level log2 ratios. In both patients, the 22q11.21 deletions were supported by contiguous regions of reduced normalized coverage consistent with heterozygous copy-number loss. No functional genetic assays, segregation testing, or orthogonal CNV confirmation by chromosomal microarray, multiplex ligation-dependent probe amplification (MLPA), quantitative polymerase chain reaction (qPCR), or copy-number variation sequencing (CNV-seq) was available. In both patients, the reported 22q11.21 deletions are therefore described as WES-derived CNV calls. The reported intervals should be interpreted as estimated CNV boundaries rather than nucleotide-level breakpoint-resolved coordinates. Source coordinates were reported on GRCh37/hg19 and mapped to GRCh38 using the Ensembl REST Assembly Mapping endpoint. Because the 22q11.21 region contains highly homologous low-copy repeats on chromosome 22 (LCR22s) that are represented differently across reference assemblies, a single GRCh37 interval may map to several discontinuous GRCh38 segments rather than one continuous block. We therefore retained the original GRCh37 coordinates for clinical interpretation and used the smallest GRCh38 interval encompassing all mapped segments only for visualization. Additional sequence-level variants of uncertain significance (VUSs), when reported, were not interpreted as causative without variant-level detail, segregation analysis, or functional validation.

## 3 Results

### 3.1 Patient 1

**Figure 1:**
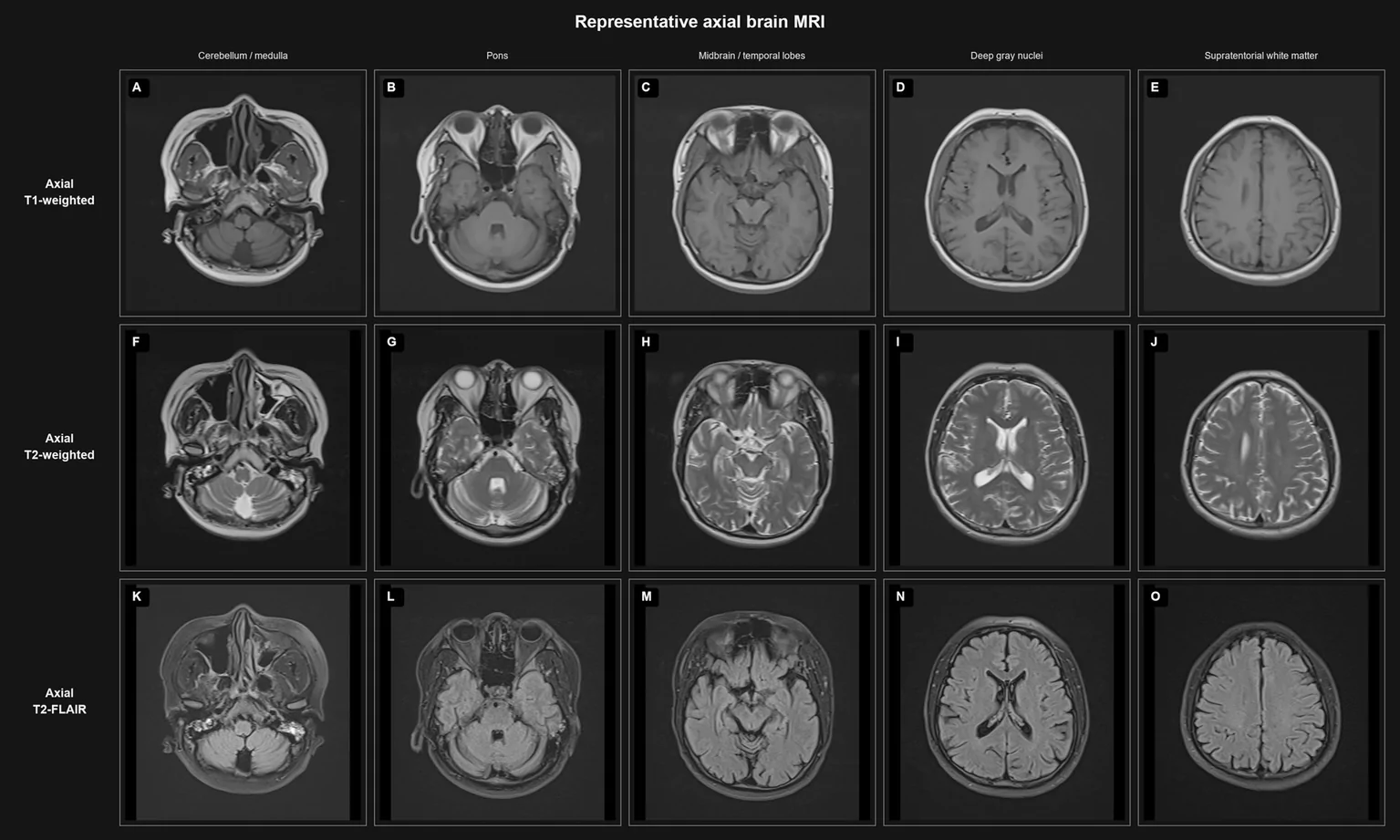
Representative axial brain magnetic resonance imaging findings in Patient 1. **(A–E)** Axial T1-weighted images showing the cerebellum, pons, midbrain, deep gray nuclei, and supratentorial regions, respectively. **(F–J)** Axial T2-weighted images corresponding to the same anatomical levels. **(K–O)** Axial fluid-attenuated inversion recovery (FLAIR) images corresponding to the same regions. Mild enlargement of the bilateral lateral ventricles and slight widening and deepening of several cortical sulci were observed. MRI, magnetic resonance imaging; FLAIR, fluid-attenuated inversion recovery.

**Figure 2:**
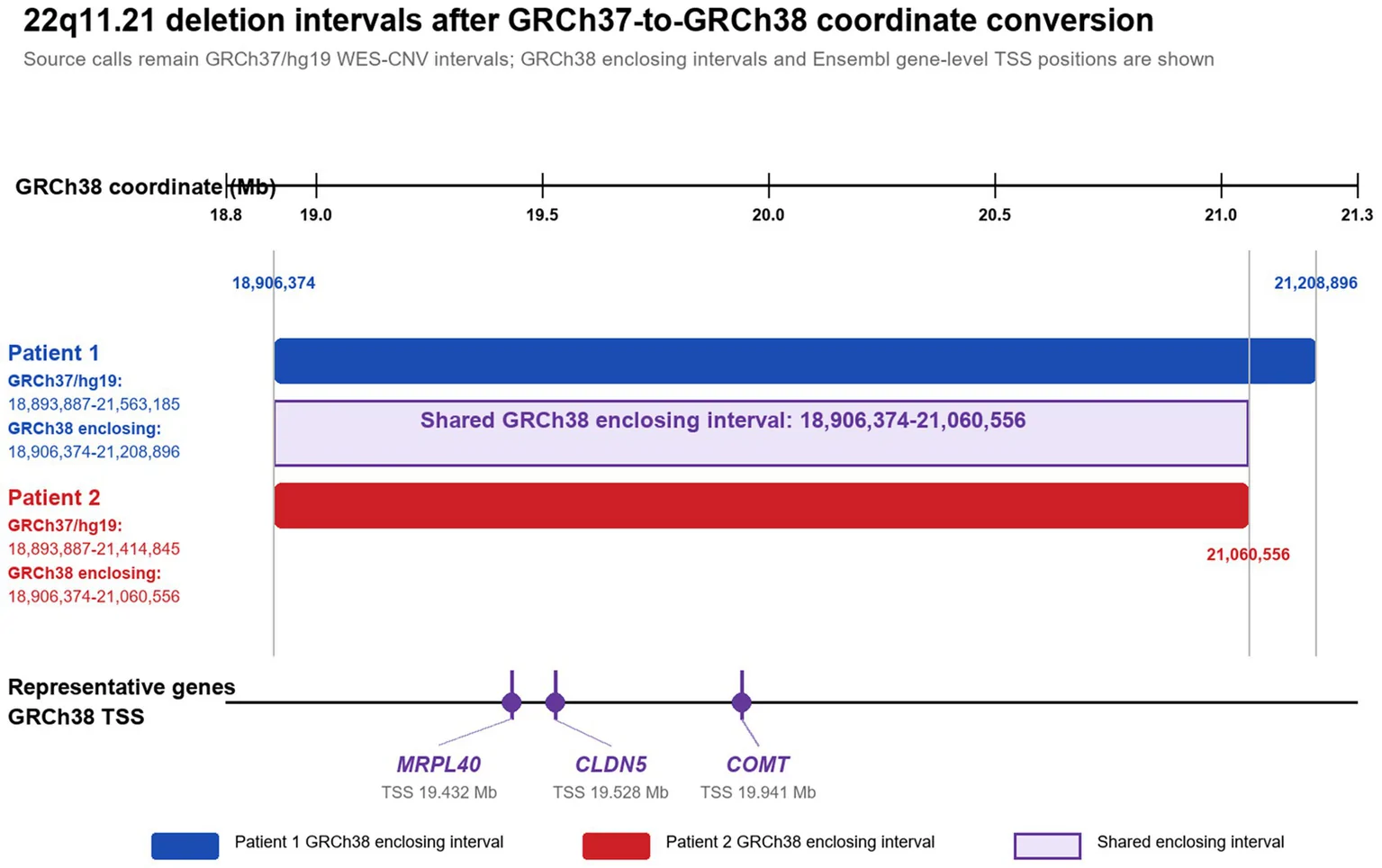
GRCh38 visualization of WES-derived 22q11.21 deletion intervals. Source calls were as follows: Patient 1, chr22:18,893,887–21,563,185; and Patient 2, chr22:18,893,887–21,414,845 (GRCh37/hg19); the corresponding GRCh38 display intervals spanning all mapped segments were as follows: Patient 1, chr22:18,906,374–21,208,896; and Patient 2, chr22:18,906,374–21,060,556. Gene-level TSS positions for *CLDN5*, *COMT*, and *MRPL40* were obtained from Ensembl GRCh38. These display intervals were used only for visualization and do not represent independently resolved CNV breakpoints. CNV, copy-number variant; GRCh37/hg19, Genome Reference Consortium Human Build 37/Human Genome 19; GRCh38, Genome Reference Consortium Human Build 38; TSS, transcription start site; WES, whole-exome sequencing.

A 29-year-old man developed progressive weakness in both lower limbs in February 2023, accompanied by an unsteady gait and difficulty initiating steps. Involuntary tremors of both lower limbs were observed during prolonged standing. Electromyography and muscle enzyme testing were unremarkable, but his gait impairment continued to progress. Cranial MRI in June 2023 (Figure 1) showed slight dilation of the bilateral lateral ventricles and widening and deepening of several sulci. During admission, WES-based CNV analysis identified a 2.67-Mb deletion in the 22q11.21 region (chr22:18,893,887–21,563,185, GRCh37/hg19; corresponding GRCh38 display interval spanning all mapped segments, chr22:18,906,374–21,208,896). Additional heterozygous VUSs in *AP4E1*, *VPS11*, *COL6A3*, *SCN1A*, and *NPHS2* were recorded in the report but were not used as diagnostic evidence (Figure 2; Supplementary Table 1).

Past medical and family history: The patient’s father reported an unremarkable birth and early growth history. The patient withdrew from school in the third grade because of poor academic performance and poor performance in physical education. His mother reportedly had a similar gait disturbance and suspected epilepsy and died at 55 years of age after sustaining a head injury during a seizure.

Neurological examination: Speech was slurred but intelligible, and responses were generally relevant. Gait was spastic and unsteady, with an involuntary lower-limb tremor during standing. Bilateral nasolabial folds were symmetrically shallow; tongue protrusion was central; pharyngeal reflexes were diminished; bilateral soft-palate elevation was absent; and the uvula was bifid with slight rightward deviation. Limb tone was mildly increased, tendon reflexes were brisk in all four limbs, ankle clonus was absent, and Babinski signs were present bilaterally. No clear upper-limb postural tremor was elicited. Rapid alternating movements of both hands were clumsy, and finger-to-nose performance was unstable and imprecise.

### 3.2 Patient 2

A 25-year-old man was admitted with progressive weakness of all four limbs, difficulty standing, and slurred speech that had progressed over 2 years. In late 2023, after a fever, he gradually developed bilateral lower-limb weakness, unsteady gait, and worsening dysarthria. In August 2024, head CT showed symmetrical bilateral basal ganglia calcification, and cranial MRI showed abnormal signal intensity in the bilateral globus pallidus. Clinicians at a previous hospital considered Fahr syndrome, but symptomatic treatment was ineffective. In May 2025, psychiatric and behavioral symptoms emerged, including irritability and obstinacy. In October 2025, WES-based CNV analysis identified a 2.52-Mb deletion in the 22q11.21 region (chr22:18,893,887–21,414,845, GRCh37/hg19; corresponding GRCh38 display interval spanning all mapped segments chr22:18,906,374–21,060,556) (Figure 2; Supplementary Table 1). A trial of carbidopa, pramipexole, and eperisone was reported to have provided no clear clinical benefit; however, the dose, duration, and objective response measures were not available.

Past medical and family history: The patient had developmental delay and learning difficulties since childhood. He underwent inguinal hernia repair at 1 year of age and developed mild dysarthria after a high fever at 6 years of age. His mother reportedly had similar motor and cognitive impairment accompanied by distinctive facial features.

**Figure 3:**
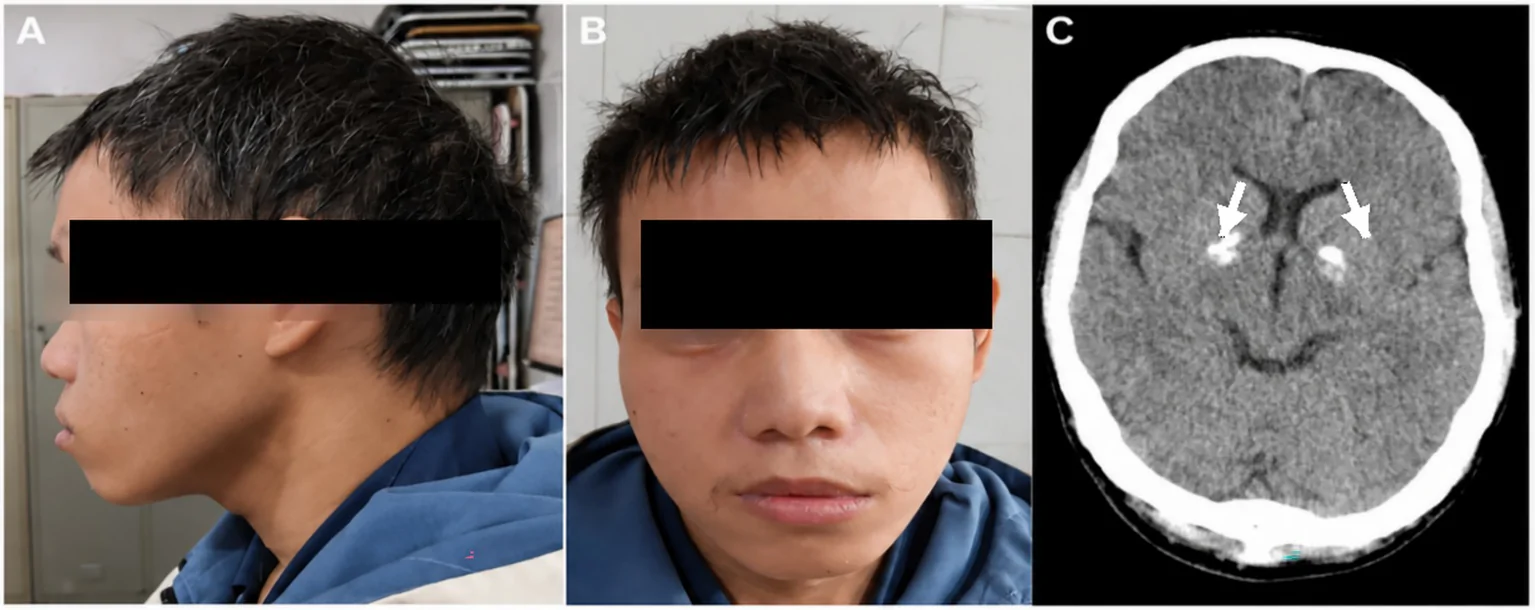
Anonymized facial photographs and basal ganglia calcification in Patient 2. **(A)** Lateral view showing left facial fullness. **(B)** Frontal view demonstrating facial asymmetry. The eye region has been masked to reduce identifiability. **(C)** Axial non-contrast head CT showing bilateral symmetric basal ganglia calcification (arrows). CT, computed tomography.

Neurological examination: He was alert, with slurred but intelligible speech, generally relevant responses, slowed reactions, and difficulty performing serial subtraction by sevens. Gait was slow and unsteady, and he was unable to squat. Bilateral nasolabial folds were symmetrically shallow; tongue protrusion was central; bilateral soft-palate elevation was slightly impaired; the uvula could not be adequately assessed because of limited cooperation; mouth opening was slightly restricted; and the tongue deviated to the left. Cogwheel rigidity was noted at both wrists, more prominently on the left. Mild to moderate lead-pipe rigidity was present at both elbows, and moderate lead-pipe rigidity was present at both knees. Rapid alternating movements of both hands were clumsy. Facial asymmetry with left-sided fullness, left-sided scoliosis, and a higher left shoulder were observed (Figures 3A,B).

Imaging and laboratory findings: Non-contrast head CT showed symmetric bilateral basal ganglia calcification (Figure 3C). Cranial MRI with diffusion-weighted imaging (DWI) showed symmetrically increased T1-weighted and decreased T2-weighted signal intensity in the bilateral globus pallidus. Magnetic resonance angiography (MRA) revealed three anterior cerebral arteries arising from both internal carotid arteries and a slender right vertebral artery. Susceptibility-weighted imaging (SWI) showed patchy mixed hyperintensity in the bilateral globus pallidus on the phase map. Serum calcium, phosphorus, and parathyroid hormone levels showed no significant abnormalities.

## 4 Discussion

### 4.1 Adult 22q11.2DS as a diagnostic pitfall in adult neurology practice

These cases show how 22q11.2DS recognized in adulthood can present in neurological care with phenotypes that resemble more familiar adult neurological diagnoses. Recent literature has broadened the motor spectrum of 22q11.2DS, with an emphasis on parkinsonism, tremor, dystonia, myoclonus, dyskinesia, pediatric motor phenotypes, and biomarker or imaging studies (1–4, 6–10). Against that background, the present cases are best framed as diagnostic-pitfall cases: Patient 1’s presentation resembled an adult pyramidal syndrome with ataxic features, such as HSP or SCA, whereas Patient 2 combined parkinsonism-mimicking rigidity and slowness with basal ganglia calcification and a Fahr-like diagnostic route. In both patients, the diagnostic direction changed when the neurological findings were considered together with developmental history, palatal or craniofacial abnormalities, psychiatric symptoms, basal ganglia calcification, and family history. The principal contribution is therefore not an expansion of the established movement-disorder spectrum but the recognition of two practical adult diagnostic pathways—HSP/SCA-like and Fahr-like presentations—that may delay the diagnosis of 22q11.2DS.

### 4.2 Pyramidal signs with ataxic features and candidate modifier variants

Patient 1 is clinically important because the dominant phenotype was progressive lower-limb spasticity with ataxic features rather than a classic congenital or psychiatric presentation. Because standardized ataxia scales, spinal imaging, and neurophysiological tract studies were not available, the most defensible description is a pyramidal syndrome with ataxic features rather than a fully characterized spastic ataxia syndrome. The additional heterozygous VUSs in *AP4E1*, *VPS11*, *COL6A3*, *SCN1A*, and *NPHS2* should not be interpreted as causative on the basis of the current evidence. They may be recorded as candidate modifier variants; however, their contribution remains unproven without variant-level details, segregation analysis, functional data, or phenotype-specific validation. The reported *SCN1A* VUS nevertheless deserves cautious clinical mention in the context of the maternal history of suspected epilepsy and seizure-related fatal head injury. *SCN1A*-related seizure disorders may show variable expressivity and incomplete penetrance (11); however, without variant-level information, electroencephalography (EEG) data, segregation analysis, or maternal genetic testing, this finding cannot be interpreted as pathogenic or as contributory in the present family. Its value in this report is therefore not mechanistic attribution but a reminder that careful seizure history-taking and longitudinal neurological monitoring should be considered when syndromic CNV disorders coexist with potentially relevant channelopathy variants. This distinction is essential: The WES-derived 22q11.21 deletion represents the principal molecular finding supporting the syndromic diagnosis, whereas the VUSs can only serve as hypotheses regarding potential phenotypic modification. A VUS should not be used as the basis for clinical decision-making (12).

### 4.3 Basal ganglia calcification and parkinsonism-mimicking presentation

Patient 2 illustrates a second diagnostic trap. Bilateral basal ganglia calcification and parkinsonism-mimicking rigidity and slowness may prompt consideration of Fahr syndrome or primary familial brain calcification, but normal serum calcium, phosphorus, and parathyroid hormone levels, together with developmental delay, learning difficulties, craniofacial features, psychiatric symptoms, and a positive family history, support consideration of a broader syndromic evaluation. The reported lack of clear benefit from carbidopa, pramipexole, and eperisone should be described cautiously because the medication dose, treatment duration, adherence data, dopamine transporter (DAT) imaging, and standardized motor scores were not available. Recent case literature also emphasizes that drug-induced or other non-degenerative parkinsonism should remain in the differential diagnosis even when 22q11.2DS is identified (6).

### 4.4 Biological plausibility and limits of mechanistic inference

Brain microvascular endothelial cells have traditionally been considered predominantly glycolytic; however, recent evidence indicates that oxidative phosphorylation also contributes to their specialized function. Endothelial Foxq1 loss disrupted endoplasmic-reticulum-to-mitochondrial calcium transfer and cristae organization, resulting in abnormal cristae, reduced oxygen consumption, and impaired ATP-linked respiration (13). These findings support a specific mitochondrial function in brain endothelium.

The deletion in Patient 2 encompasses *CLDN5*, which encodes the major tight-junction protein claudin-5. A reported 22q11.2DS case with systemic capillary leak syndrome showed greater serum-induced barrier impairment in patient-derived blood–brain barrier (BBB)-like endothelial cells than in controls (14). In complementary cellular and mouse models, 22q11.2DS was associated with BBB endothelial mitochondrial abnormalities; bezafibrate improved mitochondrial and barrier function, increased claudin-5 expression, reduced IgG leakage, and rescued social memory in mice (15). These findings remain preclinical and do not establish *CLDN5* haploinsufficiency as the sole cause of BBB dysfunction.

*De novo* heterozygous *CLDN5* missense variants have been associated with seizures, microcephaly, pontine atrophy, and brain calcifications (16). However, these variants likely exert an aberrant protein effect, and the calcification pattern differed from the symmetric basal ganglia involvement observed in our patient. Normal serum calcium, phosphorus, and parathyroid hormone levels make a common endocrine cause less likely; however, they do not establish a specific neurovascular mechanism underlying the basal ganglia calcification. *CLDN5* dosage reduction and BBB endothelial mitochondrial dysfunction therefore remain biologically plausible, but unproven, mechanisms that could contribute to the calcification and movement disorder.

## 5 Limitations

This report has several limitations. Standardized motor scales, such as the Scale for the Assessment and Rating of Ataxia (SARA), International Cooperative Ataxia Rating Scale (ICARS), or Movement Disorder Society–Unified Parkinson’s Disease Rating Scale Part III (MDS-UPDRS-III), were not available; DAT imaging was not performed; and the medication dose, treatment duration, and objective response data were unavailable for Patient 2. Orthogonal confirmation of the WES-detected CNVs, detailed variant-level interpretation of the VUSs, and parental or extended family testing were not available. Maternal testing in Patient 1 was impossible because his mother had died, and genetic testing of the mother in Patient 2 was not reported. Long-term follow-up data were also unavailable. These limitations indicate that the cases support clinical recognition and diagnostic reasoning but not a definitive mechanistic model. The CNV findings were interpreted together with the compatible clinical phenotype rather than in isolation.

## 6 Conclusion

These two cases illustrate how 22q11.2DS may remain unrecognized until adulthood when neurological care is driven by an HSP/SCA-like pyramidal syndrome with ataxic features or a Fahr-like, parkinsonism-mimicking presentation with basal ganglia calcification. The principal contribution of this report is the identification of a practical diagnostic pattern: In adults with unexplained mixed motor phenotypes, particularly when accompanied by developmental delay or learning difficulties, palatal or craniofacial abnormalities, psychiatric manifestations, basal ganglia calcification, or a suggestive family history, clinicians should consider CNV-sensitive genetic testing for 22q11.2DS. Earlier recognition may reduce diagnostic delay and facilitate multidisciplinary assessment, targeted surveillance, and genetic counseling (17) (Table 2).

**Table 2:** Clinical features that may prompt consideration of CNV-sensitive testing for 22q11.2 deletion syndrome in adult neurology practice.

Clinical category	Clinical domain	Key clinical features	Clinical relevance	Case 1 clues	Case 2 clues
Established syndromic clues	Conotruncal or aortic arch disease	Tetralogy of Fallot, truncus arteriosus, interrupted aortic arch, pulmonary atresia with VSD, right aortic arch, or vascular ring.	Classic cardiovascular manifestations of 22q11.2DS and important context for syndromic recognition.		
	Palatal or velopharyngeal abnormality	Cleft or submucous cleft palate, bifid uvula, velopharyngeal insufficiency, poor palatal elevation, or hypernasality.	Common structural or functional manifestations that may be subtle in adults and provide important syndromic context.	Absent bilateral soft-palate elevation; bifid uvula; dysarthria.	Mildly impaired bilateral soft-palate elevation; dysarthria.
	Calcium-parathyroid abnormality	Unexplained hypocalcemia or hypoparathyroidism, including stress-related episodes or hypocalcemic seizure.	A recurrent, stress-sensitive endocrine manifestation; interpret calcium together with PTH, magnesium, and clinical history.		Current calcium, phosphorus, and PTH levels were normal.
	Thymic or T-cell abnormality	Thymic aplasia or hypoplasia, T-cell lymphopenia, or otherwise unexplained cellular immunodeficiency.	A characteristic immune-developmental manifestation; document the immune phenotype and infection history.		
	Known familial deletion	A molecularly confirmed 22q11.2 deletion in a first-degree relative.	Supports targeted familial testing, parental segregation analysis, and genetic counseling.	No familial molecular testing available.	No familial molecular testing available.
Supportive multisystem clues	Neurodevelopmental or psychiatric	Developmental delay, intellectual or learning difficulty, speech-language delay, psychosis, anxiety, autism-spectrum features, or behavioral change.	Common lifelong manifestations; developmental history and change from baseline can strengthen syndromic recognition.	Poor academic performance; withdrew from school in third grade.	Childhood developmental delay and learning difficulty; later irritability and obstinacy; slowed cognition.
	Craniofacial or ENT	Characteristic craniofacial features, facial asymmetry, hearing loss, or laryngeal, tracheal, or esophageal anomaly.	May provide corroborating syndromic evidence, particularly when longstanding or accompanied by palatal or hearing abnormalities.	Symmetrically shallow nasolabial folds; dull pharyngeal reflexes.	Facial asymmetry with left-sided fullness; restricted mouth opening.
	Renal, skeletal, genitourinary, or other systemic	Renal anomaly, scoliosis, vertebral anomaly, genital anomaly, hernia, thyroid or hematologic abnormality, autoimmunity, or dysphagia.	Broadens recognition of multisystem involvement and helps distinguish a syndromic presentation from an isolated neurological disorder.		Inguinal hernia repair at the age of 1; left-sided scoliosis and higher left shoulder.
	Suggestive family history	A first-degree relative with overlapping developmental, palatal, craniofacial, psychiatric, seizure, or motor features.	May indicate an unrecognized inherited deletion and informs segregation testing and genetic counseling.	Mother had similar gait disturbance and suspected epilepsy.	Mother had similar motor and cognitive impairment with distinctive facial features.
Neurological presentations requiring syndromic reassessment	Pyramidal-ataxic phenotype	Unexplained progressive spastic gait, spastic paraparesis, bilateral pyramidal signs, or pyramidal signs with ataxic features.	An atypical adult neurological presentation that may warrant the reassessment for developmental and multisystem syndromic features.	Progressive lower-limb weakness; spastic, unsteady gait; brisk reflexes; bilateral Babinski signs; clumsy rapid alternating movements; unstable finger-to-nose testing.	
	Parkinsonism-mimicking or mixed movement disorder	Early rigidity or bradykinesia, parkinsonism, dystonia, tremor, myoclonus, or another unexplained mixed movement disorder.	Early-onset or mixed phenomenology may warrant reassessment for an underlying genomic syndrome and longitudinal movement-disorder evaluation.	Standing lower-limb tremor, without a clear parkinsonian syndrome.	Slow, unsteady gait; cogwheel and lead-pipe rigidity; slowed reactions; poor response to symptomatic therapy.
	Bilateral basal ganglia calcification	Bilateral basal ganglia calcification, with or without seizures, motor slowing, cognitive symptoms, or psychiatric symptoms.	Evaluate calcium-parathyroid metabolism and alternative causes while integrating developmental, psychiatric, and multisystem clues.		Bilateral symmetric basal ganglia calcification; abnormal globus pallidus MRI signals; normal calcium, phosphorus, and PTH.

This author-derived framework synthesizes established clinical features from published 22q11.2DS guidelines and adult neurological literature. It is intended to support clinical recognition rather than serve as a validated screening score, formal diagnostic criteria, or evidence-based testing threshold. Testing decisions should be individualized, and diagnosis requires molecular identification of a pathogenic 22q11.2 deletion. 22q11.2DS, 22q11.2 deletion syndrome; CNV, copy-number variant; ENT, ear, nose, and throat; MRI, magnetic resonance imaging; PTH, parathyroid hormone; VSD, ventricular septal defect.

Adult-neurology clinical recognition framework.

## Data Availability

The original contributions presented in the study are included in the article/Supplementary material; further inquiries can be directed to the corresponding author.
