# Supplementary material for: Adult-recognized 22q11.2 deletion syndrome in adult neurology practice: a brief report of two diagnostic pitfalls
Source: Front Neurol. 2026 Aug 31;17:1933196. doi: 10.3389/fneur.2026.1933196 (PMC13572148; doi:10.3389/fneur.2026.1933196)
Supplement: Supplementary file 1 [file Data_Sheet_1.PDF]

Clinically Relevant Multisystem Indicators in Two Patients with 22q11.2 Deletion Syndrome

Anonymized comparative summary of documented objective findings

Reading guide High/Low labels reproduce the source-record direction of abnormality. Normal and negative results are included only when directly documented.

Clinical domain	Patient 1	Patient 2
**Molecular finding**	• WES-CNV: heterozygous 2.67-Mb deletion at 22q11.21.<br>• GRCh37/hg19: chr22:18,893,887-21,563,185.<br>• Additional heterozygous VUS: AP4E1, VPS11, COL6A3, SCN1A, and NPHS2; not used as diagnostic evidence.	• WES-CNV: heterozygous 2.52-Mb deletion at 22q11.21.<br>• GRCh37/hg19: chr22:18,893,887-21,414,845.
**Syndromic and craniofacial indicators**	• Bifid uvula; absent bilateral soft-palate elevation; dull pharyngeal reflexes.<br>• Symmetrically shallow nasolabial folds.<br>• Learning impairment.<br>• Familial clue: maternal gait disorder and suspected epilepsy.	• Slightly impaired bilateral soft-palate elevation; facial asymmetry with left-sided fullness.<br>• Developmental and learning impairment.<br>• Familial clue: maternal motor and cognitive impairment with distinctive facial features.
**Neurological examination and cognition**	• Spastic, unsteady gait; lower-limb tremor while standing; dysarthria.<br>• Mildly increased limb tone; brisk tendon reflexes; bilateral Babinski signs.<br>• Clumsy rapid alternating movements; unstable finger-to-nose testing.<br>• MoCA 2/30; MMSE 7/30.	• Slow, unsteady gait; dysarthria; impaired serial subtraction.<br>• Cogwheel rigidity at both wrists; lead-pipe rigidity at elbows and knees.<br>• Clumsy rapid alternating movements.<br>• MMSE 14/30; SAS 44; SDS 39.
**Neuroimaging and vascular imaging**	• Brain MRI: mild lateral-ventricular enlargement and widened/deepened sulci reported; no focal intracranial lesion.<br>• Small periventricular T2/FLAIR hyperintense foci; left maxillary-sinus mucosal thickening.<br>• DTI: symmetric corticospinal tracts without clear compression, degeneration, interruption, or loss.<br>• MRS: no significant Lac, NAA, Cho, or Cr peak abnormality.	• Head CT: bilateral symmetric basal-ganglia calcification.<br>• MRI: symmetric globus-pallidus T1 hyperintensity and T2 hypointensity.<br>• SWI phase map: patchy mixed signal in both globus pallidus.<br>• MRA: three anterior cerebral arteries arising from both internal carotid arteries; slender right vertebral artery.
**Neurophysiology**	• EEG: mildly abnormal video EEG.<br>• Somatosensory evoked potential: prolonged right P40 latency.<br>• Nerve conduction: prolonged CMAP latencies in both common peroneal and tibial nerves; prolonged SNAP latencies in the right median and both sural nerves.<br>• Needle EMG: no major myopathic abnormality documented.	• EEG: normal.<br>• Nerve conduction and EMG: velocities and insertional activity within reference ranges; limited positive sharp waves and fibrillation potentials at rest; mixed motor-unit potentials on strong contraction.
**Cardiovascular**	• Echocardiography: mild pulmonary hypertension with mild tricuspid regurgitation; dilated coronary sinus, with possible persistent left superior vena cava.<br>• ECG: sinus tachycardia with ST-T changes.	• Echocardiography: no significant abnormality.<br>• ECG: normal.
**Endocrine, mineral, and bone**	• Calcium 1.92 mmol/L (Low); phosphorus 0.58 mmol/L (Low); potassium 2.76 mmol/L (Low).<br>• 25-hydroxyvitamin D3 5.10 ng/mL (Low).<br>• T4 64.6 nmol/L (Low); TSH 4.73 uIU/mL (High); PTH 53.94 pg/mL (within reference range).<br>• Renin-angiotensin-aldosterone indices, ACTH, and cortisol within reference ranges.	• Serum calcium, phosphorus, and PTH: no significant abnormality; PTH 21.18 pg/mL.<br>• 25-hydroxyvitamin D3 6.83 ng/mL (Low).<br>• T3 1.18 nmol/L (Low); T4 99.17 nmol/L, TSH 1.62 uIU/mL, FT3 3.43 pmol/L, and FT4 15.02 pmol/L.<br>• Bone-density assessment: osteoporosis.<br>• Renin-angiotensin-aldosterone indices, ACTH, cortisol, and testosterone within reference ranges.

Clinical domain	Patient 1	Patient 2
**Hematology and iron indices**	• Microcytic hypochromic anemia: RBC 2.02-3.84 x10^12/L (Low); hemoglobin 34-95 g/L (Low); hematocrit 14.0%-32.0% (Low).<br>• MCV 67.3 fL (Low); MCH 16.8-24.7 pg (Low); MCHC 250-298 g/L (Low); RDW-CV 20.40%-23.10% (High).<br>• Serum iron 1.04-1.86 umol/L (Low); ferritin 2.5-17.0 ng/mL (Low); UIBC 58.41-71.98 umol/L (High).<br>• Bone marrow: hypercellular marrow with active granulocytic and erythroid proliferation; M:E 1.46:1; 8 megakaryocytes with platelet aggregation.	• RBC 4.09 x10^12/L; hemoglobin 126 g/L; hematocrit 38.0% (Low); platelets 122 x10^9/L; plateletcrit 0.15% (Low).<br>• Serum iron 14.87 umol/L; UIBC 28.40 umol/L (Low); TIBC 43.27 umol/L (Low); transferrin 1.84 g/L (Low).<br>• Ferritin 739.4 ng/mL (High); haptoglobin 144.76 mg/dL; soluble transferrin receptor 2.40 mg/L.<br>• Coagulation: PT 13.3 s, INR 1.03, APTT 33.5 s, TT 16.5 s, fibrinogen 3.07 g/L.
**Immune and infectious screening**	• IgG 1,036.00 mg/dL; IgA 483.82 mg/dL (High); IgM 48.80 mg/dL (Low).<br>• T-lymphocyte subset count: no significant abnormality.<br>• Complement C3 1.16 g/L and C4 0.32 g/L.<br>• Direct Coombs test negative; broad autoantibody panel negative; rheumatoid factor and anti-parietal-cell antibody negative.<br>• Hepatitis panel negative; hepatitis B surface antibody below 2.00 mIU/mL.	• IgG 1,324.00 mg/dL; IgA 364.65 mg/dL (High); IgM 108.77 mg/dL.<br>• Interleukin panel: no significant abnormality.<br>• Hepatitis, HIV, and syphilis screening negative; hepatitis B surface antibody below 2.00 mIU/mL.<br>• Antistreptolysin O 57.31 IU/mL; ESR 12 mm/h.
**Metabolic and hepatobiliary**	• Uric acid 463-530 umol/L (High).<br>• Lactate 3,677.4-4,090.7 umol/L (High); pyruvate 169.31 umol/L (High).<br>• ALT 59 U/L (High); AST 121 U/L (High); mitochondrial AST 34.3 U/L (High); LDH 258 U/L (High).<br>• Triglycerides 2.29 mmol/L (High); cholinesterase 4,025 U/L (Low).<br>• Abdominal ultrasonography: no focal liver or splenic lesion; no biliary calculus or dilatation.	• Uric acid 329 umol/L.<br>• Lactate 1,185.4 umol/L; pyruvate 83.55 umol/L.<br>• ALT 19 U/L, AST 21 U/L, GGT 12 U/L, ALP 64 U/L, total bilirubin 7.4 umol/L, albumin 46.9 g/L.<br>• Ultrasonography: post-cholecystectomy state; no biliary calculus or dilatation.<br>• Copper 13.86 umol/L, ceruloplasmin 307.4 mg/L, and copper oxidase 0.636 OD.
**Renal and urinary**	• Renal ultrasonography: no focal renal lesion.<br>• Urinalysis: leukocyte esterase weakly positive; leukocytes 29.12/uL and 5.20/HPF (High); fungal smear negative.	• Ultrasonography: right renal calculus.<br>• Urinalysis: urobilinogen +1 and bilirubin +1; protein, glucose, occult blood, leukocyte esterase, and nitrite negative; no urinary-tract infection signal.<br>• Urea 5.82 mmol/L; creatinine 72 umol/L.
**Respiratory and thymic**	• Chest CT: possible small right-apical pulmonary nodule; increased and disordered bilateral lung markings.<br>• Thymic CT: no definite space-occupying lesion.	• Chest radiography: no significant parenchymal lung lesion.<br>• Thymic CT: no abnormal density.
**Musculoskeletal**	• Cervical MRI: C5-C6 disc bulge and bilateral ligamentum-flavum thickening.<br>• Thoracic MRI: T11 vertebral endplate inflammation.	• Left-sided scoliosis with higher left shoulder.<br>• Spinal imaging: scoliosis and degenerative changes; lumbar-disc protrusion documented.<br>• Bone-density assessment: osteoporosis.
